# Nuclear-encoded cytochrome c oxidase subunit 4 regulates BMI1 expression and determines proliferative capacity of high-grade gliomas

**DOI:** 10.18632/oncotarget.3015

**Published:** 2015-01-20

**Authors:** Claudia R. Oliva, Tahireh Markert, G. Yancey Gillespie, Corinne E. Griguer

**Affiliations:** ^1^ Department of Neurosurgery, University of Alabama at Birmingham, Birmingham, Alabama; ^2^ Center for Free Radical Biology, University of Alabama at Birmingham, Birmingham, Alabama

**Keywords:** Cytochrome c oxidase, BMI1, COX4, glioma, proliferation

## Abstract

Nuclear-encoded cytochrome c oxidase subunit 4 (COX4) is a key regulatory subunit of mammalian cytochrome c oxidase, and recent studies have demonstrated that COX4 isoform 1 (COX4-1) could have a role in glioma chemoresistance. The Polycomb complex protein BMI1 is a stem cell regulatory gene implicated in the pathogenesis of many aggressive cancers, including glioma. This study sought to determine if COX4 regulates BMI1 and modulates tumor cell proliferation. Using The Cancer Genome Atlas database and a retrospective data set from patients with *glioblastoma multiforme*, we found that BMI1 expression levels positively correlated with COX4-1 expression and overall survival. Whereas COX4-1 promoted cell growth by increasing BMI1 expression, COX4-2 inhibited cell growth even in cells overexpressing BMI1. We also demonstrate that COX4-1 attenuates mitochondrial reactive oxygen species (ROS) production, which is required for COX4-1-mediated effects on BMI1 expression and cell proliferation. Notably, mice bearing COX4-1-expressing glioma cell xenografts quickly developed invasive tumors characterized by the presence of multiple lesions positive for Ki-67, BMI1, and COX4-1, whereas mice bearing COX4-2-expressing xenografts rarely developed tumors by this point. COX4-1 also promoted the self-renewal of glioma stem-like cells, consistent with the reported role of BMI1 in stem cell growth. Taken together, these findings identify a novel COX4-1-mitochondrial ROS axis, in which differential expression of COX4 isoforms regulates mitochondrial ROS production and controls *BMI1* expression.

## INTRODUCTION

Cytochrome c oxidase (CcO, complex IV; EC 1.9.3.1) is the terminal enzyme of the mitochondrial respiratory chain (electron transport chain, ETC) that catalyzes the transfer of electrons from cytochrome *c* to oxygen (O_2_). CcO is a complex enzyme consisting of 13 subunits, three of which are encoded by mitochondrial DNA (mtDNA) and perform the catalytic function of CcO, and 10 of which are nuclear-encoded and provide the regulatory function [1, 2]. Several studies have presented CcO as an essential regulator of overall ETC activity in mammalian cells; decreased CcO activity decreases ATP production, whereas increased CcO activity augments the electron flux capacity of the ETC, leading to more efficient mitochondrial coupling and reduced production of reactive oxygen species (ROS) [3–6]. Expression, assembly, and activity of CcO are highly regulated, and intrinsic biochemical parameters of CcO were shown to be tissue-specific due to differential isoform expression [7, 8]. We recently demonstrated that elevated CcO activity is a characteristic of chemoresistant glioma. Moreover, higher CcO activity is associated with poor overall survival (OS) and progression-free survival (PFS) in patients with newly diagnosed *glioblastoma multiforme* (GBM) [9]. Indeed, subsets of patients with primary GBM (25%–30% of the patient population) have extremely low OS (6.3 months).

BMI1, a member of the Polycomb family of transcriptional repressors that mediate gene silencing by regulating chromatin structure, is essential for self-renewal and has been implicated in the maintenance of stem cells in several tissues [10–13]. Notably, BMI1 has been reported to be associated with the progression, recurrence, and chemoresistance of various types of cancer cells [14–18]. However, little is known about how BMI1 is regulated in glioma cells. Here, we report that COX4-1 and BMI1 are co-expressed in highly proliferative human GBM tumors and highly enriched in tumor-initiating stem cells. We provide evidence that COX4-1 controls BMI1 expression via a redox mechanism. When implanted in the brains of nude mice, COX4-1-bearing cells developed multi-centric lesion tumors. Thus, our findings provide a molecular mechanism explaining how COX4-1 regulates BMI1 expression and reveal the biological impact of COX4-1 and mitochondrial function on the development of a subset of GBMs with a worse prognosis.

## RESULTS

### COX4-1 expression correlates with BMI1 expression and overall survival in patients with high-grade GBM

**Figure 1:**
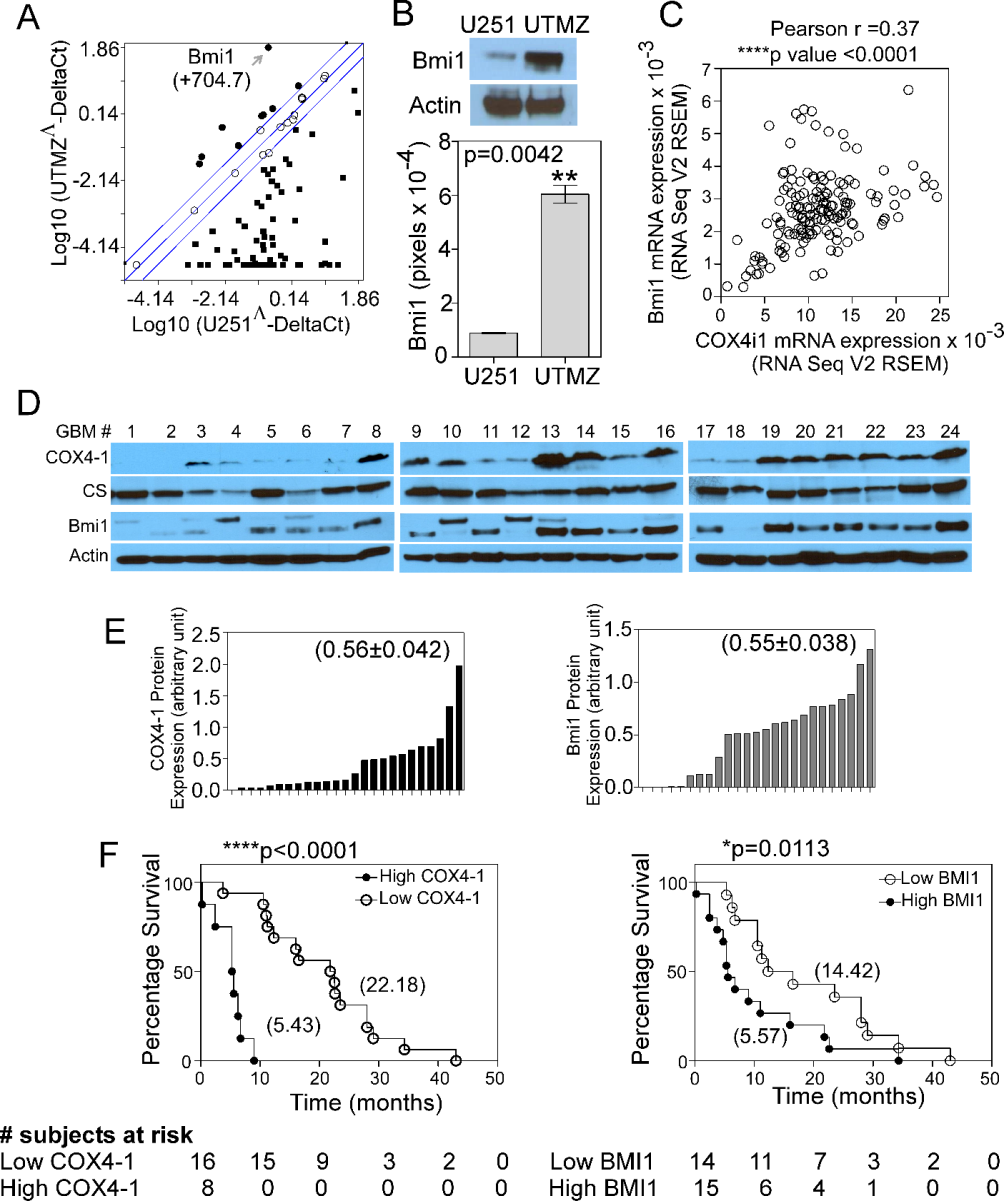
COX4-1 correlates with BMI1 expression and low OS of patients in primary GBM **(A)** Scatter plot of PCR array data showing relative gene expression levels in UTMZ cells relative to U251 cells. Genes upregulated by more than 2-fold are shown in black circles, genes downregulated by more than 2-fold are shown in black squares. Arrow shows the data point representing *BMI1*. **(B)** Representative western blot (top) and quantitative analysis (bottom graph) showing the relative BMI1 expression levels in U251 and UTMZ cells. **(C)** Analysis of RNA-sequencing data provided by TCGA depicting co-expression of *COX4I1* mRNA and *BMI1* mRNA in patients with high-grade GBM. **(D)** Representative western blots depicting COX4-1 and BMI1 expression in a panel of 24 primary human GBM tumors. **(E)** Quantification of relative band intensities in (D) Numbers in parentheses indicate the mean value from all tumors. **(F)** OS for patients with high and low tumor expression levels of COX4-1 (*P* < 0.0001 by the log-rank test; hazard ratio for death in patients with high tumor COX4-1 expression, 54.99; 95% CI, 11.02 to 274.3) or BMI1 (*P* = 0.0113 by the log-rank test; hazard ratio for death in patients with high tumor BMI1 expression, 2.59; 95% CI, 2.107 to 3.073). Numbers in parentheses indicate the median survival time for each group.

U251-MG glioma cells express the COX4-2 isoform predominantly, whereas temozolomide (TMZ)-resistant UTMZ glioma cells derived from U251-MG cells by drug selection express the COX4-1 isoform predominantly and correlated with a more aggressive phenotype. [4]. These observations prompted us to further examine the mechanism of COX4-1-associated glioma cell growth. We used the Human Cancer PathwayFinder™ RT^2^ Profiler™ PCR Array to ascertain changes in tumor-promoting genes occurring in COX4-1-expressing cells that could be responsible for the pro-tumorigenic effects. Out of the 84 genes explored, 71 genes were differentially modulated by more than 2-fold in COX4-1-expressing (UTMZ) glioma cells versus COX4-2-expressing (U251) glioma cells. Out of these 71 genes, nine were upregulated and 62 were downregulated (Figure 1A). *BMI1*, the most highly upregulated (approximately 704-fold) gene in the array, was upregulated 6-fold at the protein level in UTMZ cells compared with U251 cells (Figure 1B, *p* = 0.0042).

**Figure 2:**
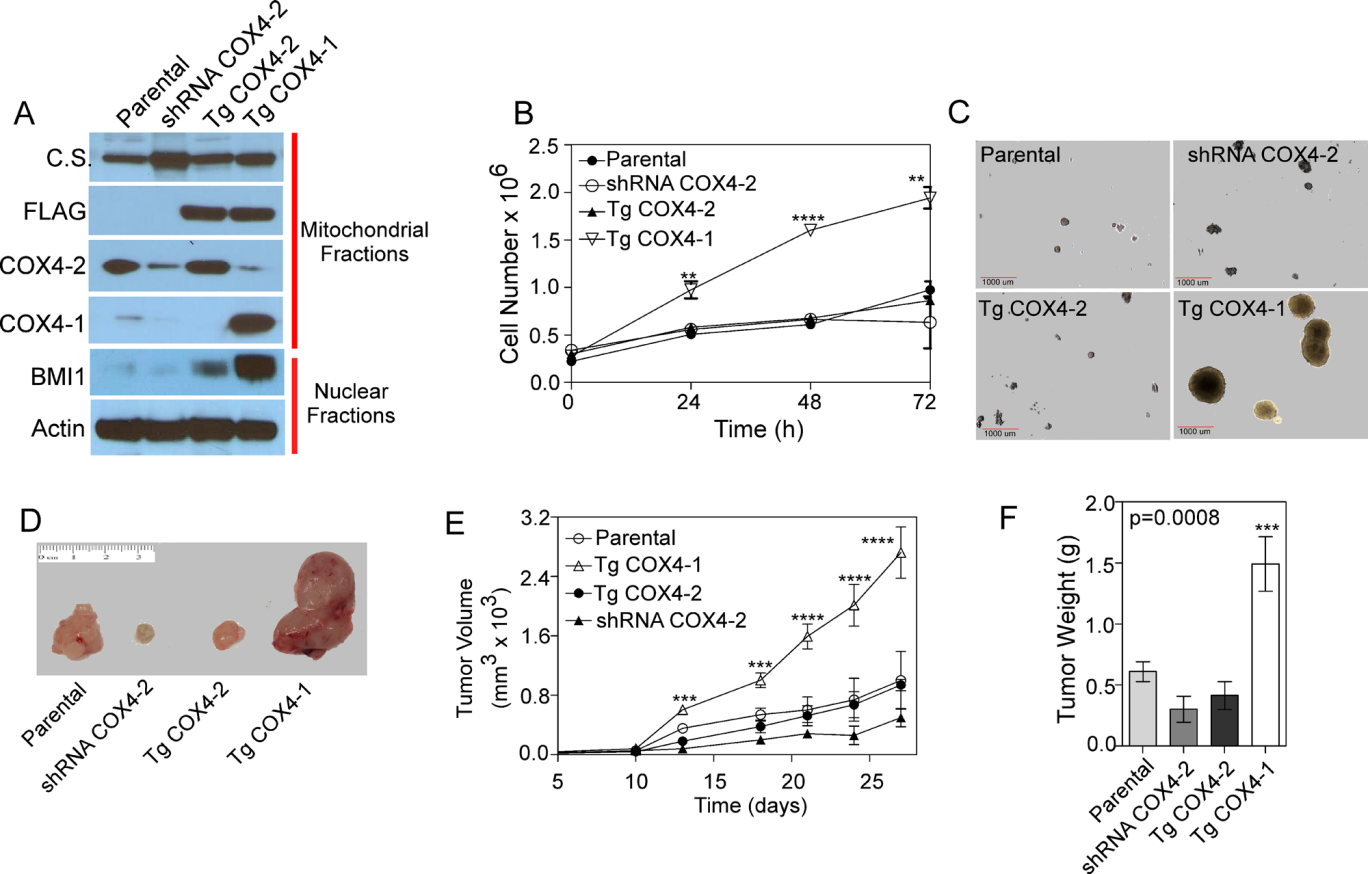
COX4-1 drives proliferative capacity in human glioma cells **(A)** COX4-1 and COX4-2 constructs ( pCMV6-COX4-1-FLAG and pCMV6-COX4-2-FLAG) were transfected into U251-COX4-2 depleted cells to create U251-TgCOX4-1 and U251-TgCOX4-2 stable cell lines. Expression of COX4 isoforms and BMI1 was detected in each cell line by western blot analysis. Citrate synthase (CS) expression is shown as mitochondrial loading control and actin expression is shown as nuclear loading control. **(B)** Proliferation rates of each cell line. **(C)** Representative pictures of clonogenic assays with each cell line, showing anchorage-independent cell growth. **(D)** Representative images of tumors from athymic nude mice inoculated with the cell lines. Tumors were excised 4 weeks after inoculation. **(E)** Analysis of tumor volumes in mice over the course of the experiment. **(F)** Comparison of tumor weights upon excision. Graphs represent the average from triplicate determinations from at least three independent experiments.

By analyzing data from The Cancer Genome Atlas (TCGA) (http://cancergenome.nih.gov/), we found that mRNA expression of the *COX4I1* gene, which encodes COX4-1, is significantly correlated with the expression of *BMI1* mRNA in patients with high-grade GBM (Pearson correlation, *p* < 0.0001) (Figure 1C). No correlation was found between the expression levels of *COX4I1* mRNA and *BMI1* mRNA in patients with low-grade GBM or between those of *COX4I2* mRNA and *BMI1* mRNA in patients with high-grade GBM (data not shown). We then examined COX4-1 and BMI1 expression levels by western blot analysis in a panel of 24 GBM tumors (Figure 1D) and found that high COX4-1 expression positively correlated with high BMI1 expression (Figure 1E). Some of the tumor samples displayed up-shifted migration bands for BMI1 (tumor samples numbers 4, 10 and 12). It is possible that those bands represent a phosphorylated form of BMI1 as previously described [19, 20]. Patient's survival data were ranked based on their tumor expression of COX4-1 (Figure 2E). Patients with COX4-1 values over the mean value of the population were defined as “high COX4-1” and patients with COX4-1 values below the mean value of the population were defined as “low COX4-1.” Kaplan-Meier survival analysis and log-rank significance tests performed for these two groups showed that high COX4-1 expression correlated with worst patient prognosis (Figure 1F). There was a significant difference in OS between patients whose tumors had high or low COX4-1 expression. High COX4-1 was detected in 8 patients (33%) and was associated with poor OS. The median OS among patients with low COX4-1 was 22.18 months, compared with 5.43 months among patients with high COX4-1 expression (*p* < 0.0001 by log-rank test). High BMI1 expression in tumors was also associated with shorter OS. The median OS among patients with low BMI1 was 14.41 months compared with 5.5 months among patients with high BMI1 expression (*p* = 0.0113 by log-rank test) (Figure 1F). These findings confirm the mRNA studies in primary GBM from TCGA and suggest roles for COX4-1 and BMI1 in GBM progression.

### COX4-1 regulates BMI1 expression and is essential for GBM proliferation

To decipher the functional properties of COX4 isoforms in GBM proliferation, U251 cells depleted of endogenous COX4-2 were stably transfected with expression vectors encoding either FLAG-epitope-tagged COX4-1 (U251-TgCOX4-1) or FLAG-epitope-tagged COX4-2 (U251-TgCOX4-2). Immunoblot analysis of isolated mitochondria revealed that the COX4 isoforms were expressed at high levels in mitochondrial fractions (Figure 2A). BMI1 expression analysis revealed significantly elevated BMI1 levels in U251-TgCOX4-1 cells compared with U251-TgCOX4-2 and parental U251 cells (Figure 2A). Significantly more cell proliferation was observed in U251-TgCOX4-1 cells compared with U251-TgCOX4-2 and U251 cells (*p* < 0.005 and *p* < 0.0001, respectively; Figure 2B), and more anchorage-independent growth was observed in U251-TgCOX4-1 cells (Figure 2C). To determine the effects of COX4-1 expression *in vivo*, equivalent numbers of parental U251, U251-TgCOX4-1, U251-TgCOX4-2, or U251-shRNA-COX4-2 cells were inoculated subcutaneously into the flanks of athymic nude mice. Mice injected with U251-TgCOX4-1 cells developed tumors significantly larger in volume (3-fold, *p* < 0.0001) and weight (4-fold, *p* = 0.0008) compared with U251-TgCOX4-2 cell-inoculated counterparts (Figure 2D–2F). These results suggest that COX4-1 may have oncogenic properties in GBM and promote tumorigenesis.

**Figure 3:**
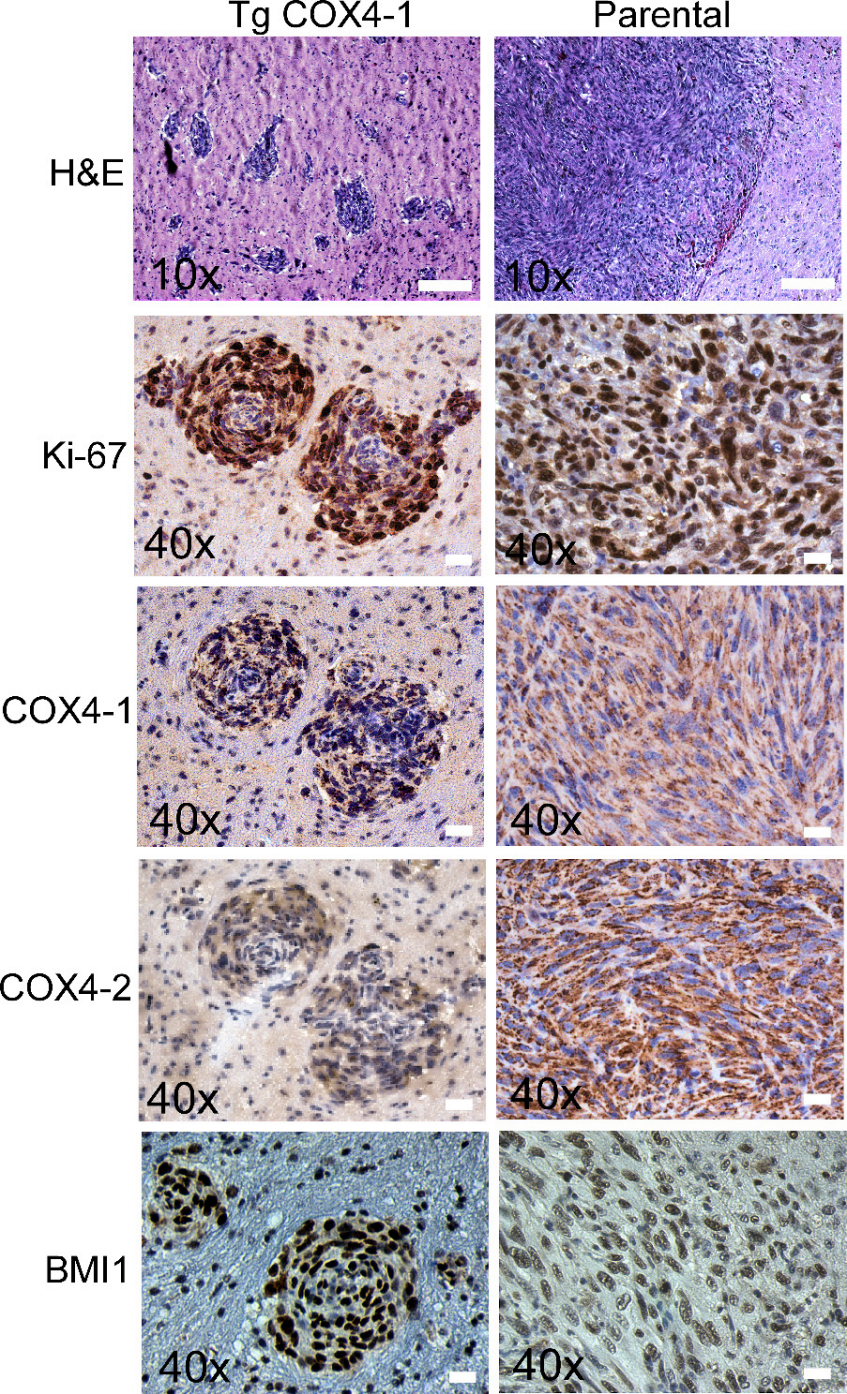
COX4-1 expression correlates with multicentric distribution of GBM within the brain parenchyma Representative images of tumors resulting from intracranial implantation of U251 and U251-TgCOX4-1 glioma cells, stained for **(A)** H&E, **(B)** Ki-67, **(C)** COX4-1, **(D)** COX4-2, and **(E)** BMI1. Scale bar, 100 μm.

Next, we examined the effects of COX4 isoform expression in an orthotopic mouse model. U251-TgCOX4-1, U251-TgCOX4-2, U251-shRNA-COX4-2, or parental U251 cells (5 × 10^5^ cells each) were inoculated in the caudate putamen in the striatal area of the brain of immunocompromised mice, and mice were sacrificed 30 days later. No tumors were detected in the brains from mice inoculated with U251-shRNA-COX4-2 cells or U251-TgCOX4-2 cells, indicating a significantly slower progression of these tumors *in vivo*. However, mice bearing U251-TgCOX4-1 cells developed invasive tumors characterized by multiple tumor loci throughout the entire brain parenchyma. In comparison, brains with parental U251 tumors displayed only a single lesion (Figure 3). Immunostaining of the multifoci showed markedly higher COX4-1 and BMI1 levels, along with substantially more Ki-67 staining that associated with multiple tumor loci in U251-TgCOX4-1 xenografts compared with parental controls (Figure 3), suggesting that COX4-1 expression promotes *in vivo* tumor cell proliferation.

### Proliferation potential of COX4-1 cells is BMI1-dependent

**Figure 4:**
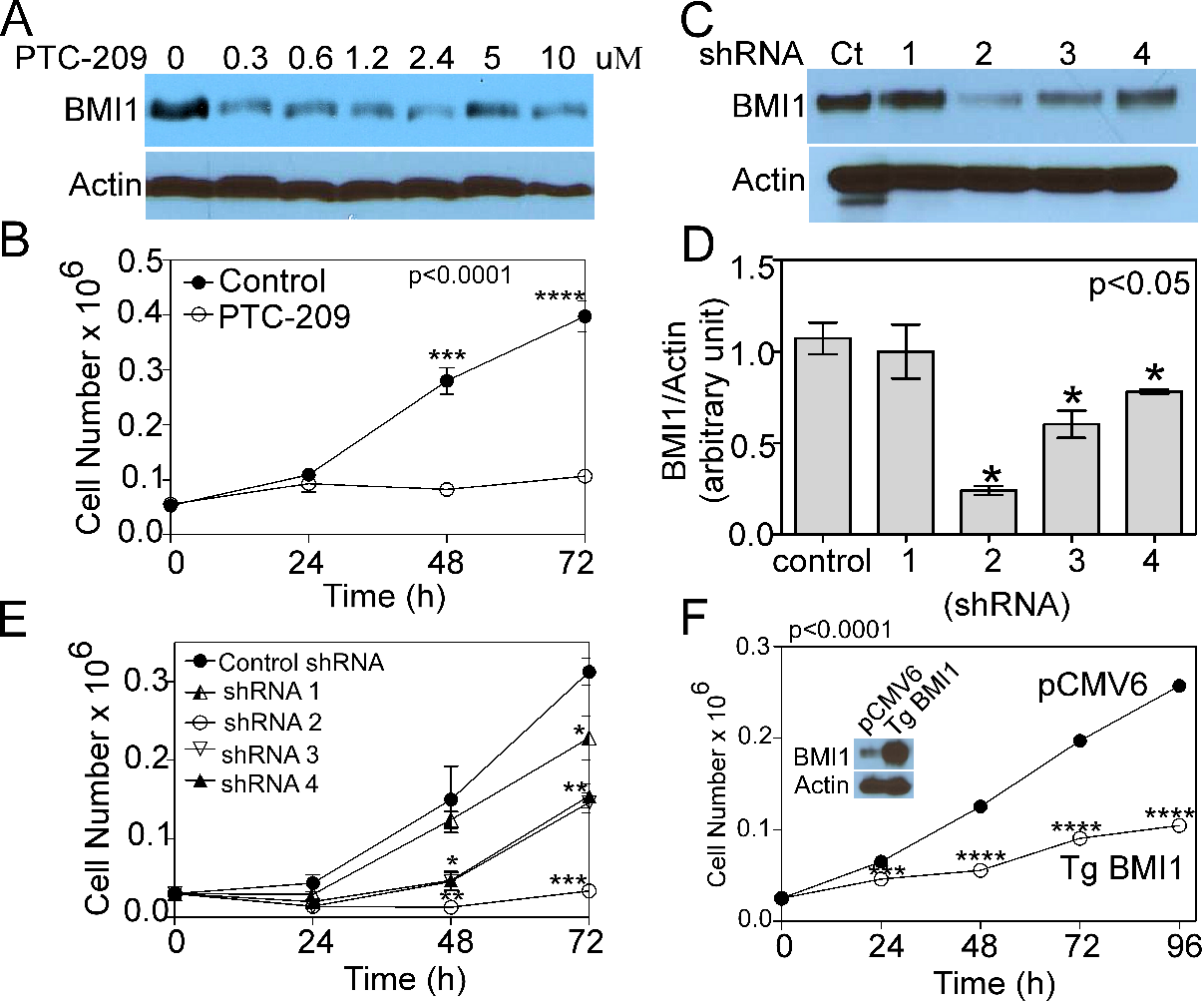
COX4-1 and BMI1 co-expression is required to promote cell proliferation **(A)** Representative western blot depicting BMI1 expression in nuclear extracts of U251-TgCOX4-1 cell following 24-h PTC-209 treatment (0–10 μM). **(B)** Cell proliferation in control and PTC-209-treated (5 μM) U251-TgCOX4-1 cells. **(C)** Representative western blot depicting BMI1 expression in U251-TgCOX4-1 cells expressing shRNA control or one of four different vectors expressing shRNA against BMI1. **(D)** Quantification of the relative expression levels of BMI1 detected in (C). **(E)** Cell proliferation in clones expressing shRNA against BMI1. **(F)** Representative western blot depicting BMI1 expression levels (inset) and the cell proliferation rates of control and pCMV6-BMI1-transfected U251 cells. Graphs represent the average from triplicate determinations from at least three independent experiments.

To gain insight into the mechanism by which COX4 isoform expression regulates tumor proliferation and phenotypic changes, we determined whether reduction of BMI1 levels affects the growth of glioma cells. Treatment of U251-TgCOX4-1 cells with PTC-209, a small-molecule BMI1 inhibitor [21], reduced BMI1 expression (Figure 4A) and cell proliferation (Figure 4B). To more directly establish a role for BMI1 in cell proliferation, we generated U251-TgCOX4-1 cells with BMI1 knockdown. A total of four different lentivirus-encoded shRNAs for BMI1 were used to knock down BMI1, with each shRNA yielding different results. Clone 1 shRNA-infected cells expressed *BMI1* mRNA levels similar to the scramble-shRNA-control cells and showed similar rates of proliferation. Clone 2 shRNA-infected cells (<80% knockdown of *BMI1*) progressively lost the ability to grow *in vitro*, and cells expressing shRNA clones 3 and 4 (<40–60% knockdown of *BMI1*) displayed a 2-fold reduction in cell proliferation compared with cells expressing shRNA-control (Figure 4C–4E). To investigate the effect of BMI1 on the aggressiveness of COX4-2 glioma cells, U251 cells stably overexpressing BMI1 were established (Figure 4F inset). The proliferation rate of cells overexpressing BMI1 was 2.5-fold lower than that of control cells (*p* < 0.0001) (Figure 4F). Collectively, these data indicate that GBM cells require both COX4-1 and BMI1 expression to promote cell growth *in vitro*.

**Figure 5:**
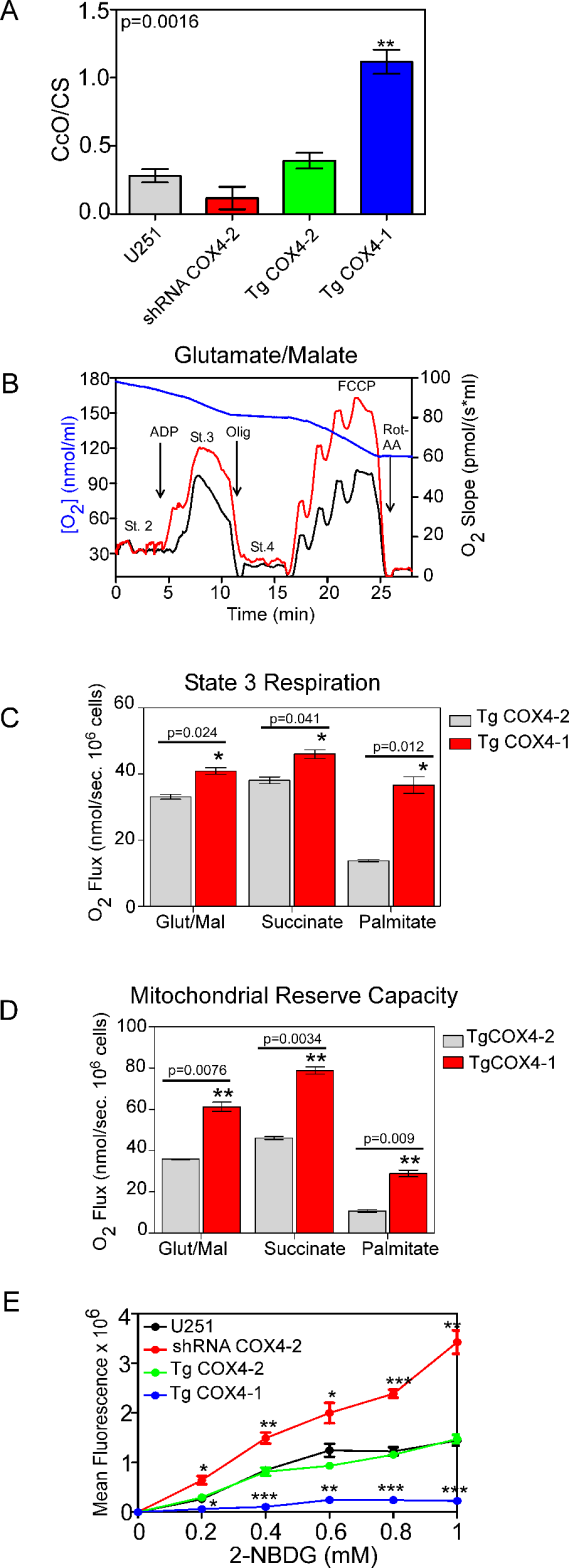
COX4-1 expression induces changes in mitochondrial function **(A)** Relative activity of CcO normalized to citrate synthase (CS) activity. **(B)** Oxygen consumption rates were determined using a respirometer. Representative traces of cellular respiration rates of U251 (black) and U251-TgCOX4-1 (red) cells (blue line, oxygen concentration). **(C)** Kinetic characterization of glutamate/malate, succinate, and fatty acid-dependent respiration of U251-TgCOX4-1 and U251-TgCOX4-2 cells. **(D)** Kinetic characterization of FCCP-dependent respiration in U251-TgCOX4-1 and U251-TgCOX4-2 cells. **(E)** Dose-response analyses of glucose uptake in cell lines expressing different COX4 isoforms. Graphs represent the average from triplicate determinations from at least three independent experiments.

Because it was previously reported that BMI1 is involved in maintaining mitochondrial function and regulating cellular metabolism in mouse thymocytes [22], we analyzed the mitochondrial function in U251-TgCOX4-1 cells after pharmacologic downregulation of BMI1. Compared with parental U251 or U251-TgCOX4-2 cells, U251-TgCOX4-1 cells had higher CcO activity Figure 5A, mitochondrial respiration Figure 5B and 5C, and spare capacity Figure 5D and lower glucose uptake Figure 5E, suggesting a switch to a more OXPHOS-dependent metabolism. U251-TgCOX4-1 cells treated with 5 μM PTC-209 to silence BMI1 displayed mitochondrial oxidative capacity (states 2, 3, and 4) and FCCP-dependent respiration similar to those of control cells (Table 1). We used FCCP to uncouple mitochondrial electron transport through complexes I to IV from phosphorylation (complex V) with the aim of evaluating metabolic flux control by the phosphorylation system over the electron transport capacity. Consistent with our intact cellular respiration measurements, mitochondrial complex activities were similar in mitochondria purified from control and BMI1-depleted cells (Table 1). Collectively, these data indicate that COX4-1 regulates mitochondrial function in glioma cells independently of BMI1 expression.

**Table 1:** Effect of PTC-209 on mitochondrial respiration rates and mitochondrial complexes activities

O_2_ consumption, O_2_ slope [pmoles/(sec.10^6^ cells)^a^]					Mitochondrial Complexes Activities/Citrate Synthase^b^				
	State 2	State 3	State 4_olig_	FCCP	Rot/AA	CI/CS	CII-III/CS	CcO/CS	CV/CS
**Control**	43.28 ± 2.1	130.2 ± 9.5	8.5 ± 0.6	137.7 ± 6.2	3.06 ± 0.2	1.9 ± 0.05	1.23 ± 0.06	1.04 ± 0.07	0.8 ± 0.02
**PTC-209**	40.57 ± 3.5	127.5 ± 7.1	7.86 ± 0.8	135.2 ± 8.7	3.08 ± 0.4	1.8 ± 0.08	1.15 ± 0.01	1.2 ± 0.12	0.77 ± 0.03

a Values of representative O_2_ consumption rates are normalized to the amount of cells added to the chamber.

b Mitochondrial complexes activities are normalized by citrate synthase activity

### COX4-1 regulates BMI1 expression by decreasing mitochondrial ROS production

**Figure 6:**
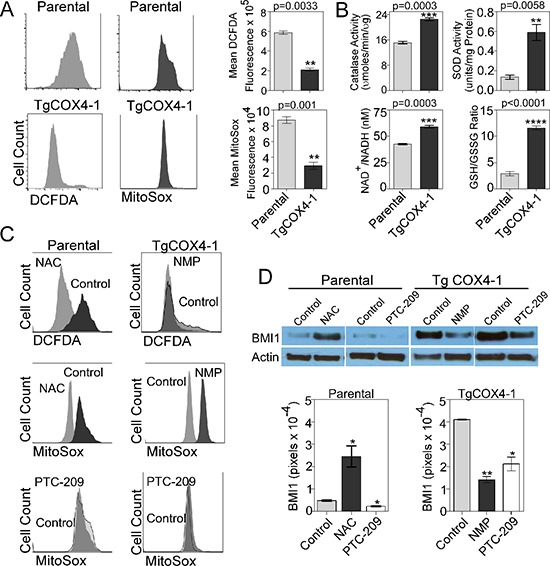
Mitochondrial ROS regulates BMI1 expression **(A)** Representative histograms from flow cytometric analysis of total cellular ROS (left, DCFDA fluorescence) and mitochondrial ROS (right, MitoSOX fluorescence) in parental and U251-TgCOX4-1 cells. Bar graphs provide quantitative analysis of fluorescence intensity. **(B)** Quantitative graphs showing the relative levels of catalase activity, superoxide dismutase activity, NAD^+^/NADH ratio, and GSH/GSSG ratio in U251-TgCOX4-1 cells. **(C)** Representative histograms from flow cytometric analysis of total cellular or mitochondrial ROS production in U251-TgCOX4-2 cells treated with NAC (300 μM) or PTC-209 (5 μM) (left) and in U251-TgCOX4-1 cells treated with NMP (10 μM) or PTC-209 (right). **(D)** Representative western blots depicting BMI1 expression in the nuclear extracts of parental cells or U251-TgCOX4-1 cells after treatment with NAC or PTC-209 for 24 h (top) and quantitative analysis of expression levels (bottom). Bars represent the average from triplicate determinations from at least three independent experiments.

The higher efficiency of mitochondrial metabolism (Figure 5) in U251-TgCOX4-1 cells might be reflected in lower mitochondrial ROS production. To address the effect of COX4-1 expression on ROS production, U251 parental and U251-TgCOX4-1 glioma cells were analyzed for basal intracellular ROS levels. The mean values of 2′,7′–dichlorofluorescein diacetate (DCFDA) and MitoSOX™ Red fluorescence were used to calculate the fold difference in cellular and mitochondrial ROS levels, respectively. Flow cytometric analysis revealed that U251-TgCOX4-1 cells displayed significantly lower levels of cellular (2.8-fold, *p* = 0.0033) and mitochondrial ROS (3-fold, *p* = 0.001) (Figure 6A). Catalase (CAT, EC 1.11.1.6) and superoxide dismutase (SOD, EC 1.15.1.1) are ubiquitous antioxidant enzymes with critical roles in removing cellular peroxides. U251-TgCOX4-1 cells maintained a 2-fold higher level of CAT activity (*p* = 0.0003) and a 4.4-fold higher level of SOD activity (*p* = 0.0058) compared with parental U251 cells (Figure 6B). We next investigated the intracellular ratios of reduced glutathione (GSH) to oxidized glutathione disulfide (GSSG) to determine if COX4-1 overexpression influences this redox couple, which would alter the cellular capacity to resist oxidative stress. Indeed, U251-TgCOX4-1 cells maintained a higher GSH/GSSG ratio (10.86) than parental U251 cells (2.87) did (Figure 6B).

The balance between the oxidized and reduced forms of nicotinamide adenine dinucleotide (NAD), measured by the NAD^+^/NADH ratio, is also an important indicator of the redox state of a cell, reflecting both the metabolic activity and the health of cells. Increased mitochondrial activity, reduced glycolytic flux, and accelerated flow of electrons in the ETC, as evident in mitochondria from U251-TgCOX4-1 cells (Figure 5), decreases the likelihood of superoxide formation and should be accompanied by an increased NAD^+^/NADH ratio. Consistent with such a mechanism, the NAD^+^/NADH level was 40% higher (*p* = 0.0003) in U251-TgCOX4-1 cells than in parental cells (Figure 6B).

We next assessed whether the difference in mitochondrial ROS levels in U251-TgCOX4-1 and U251-TgCOX4-2 cells contributes to the regulation of BMI1 expression. Treatment of parental U251 cells with the antioxidant scavenger N-acetylcysteine (NAC) for 24 h reduced cellular and mitochondrial ROS to levels similar to those in U251-TgCOX4-1 cells (Figure 6C). This decrease in ROS production was accompanied by a significant increase (5.5-fold, *p* = 0.015) in the expression of BMI1 (Figure 6D). Previous studies demonstrated that treatment of glioma cells with N-methyl mesoporphyrin IX (NMP), an inhibitor of ferrochelatase, blocks the activity of CcO [4]. To examine whether increased levels of mitochondrial ROS affect BMI1 expression, we treated U251-TgCOX4-1 cells with NMP for 48 h. NMP treatment increased mitochondrial ROS but did not appreciably alter total ROS production (Figure 6C). This increase in mitochondrial ROS was accompanied by a significant downregulation in the expression of BMI1 (2.9-fold, *p* = 0.0026) (Figure 6D). Finally, we tested the effect of PTC-209 on ROS production. PTC-209 reduced the expression of BMI1 by 2.2- and 1.9-fold in parental cells and U251-Tg-COX4-1 cells, respectively. However, no changes in mitochondrial ROS production were detected. Thus, in agreement with previous results (Table 1), pharmacological downregulation of BMI1 had no effect on mitochondrial function in glioma cells.

### COX4-1 promotes neurosphere formation and upregulation of stem cell markers in GBM

**Figure 7:**
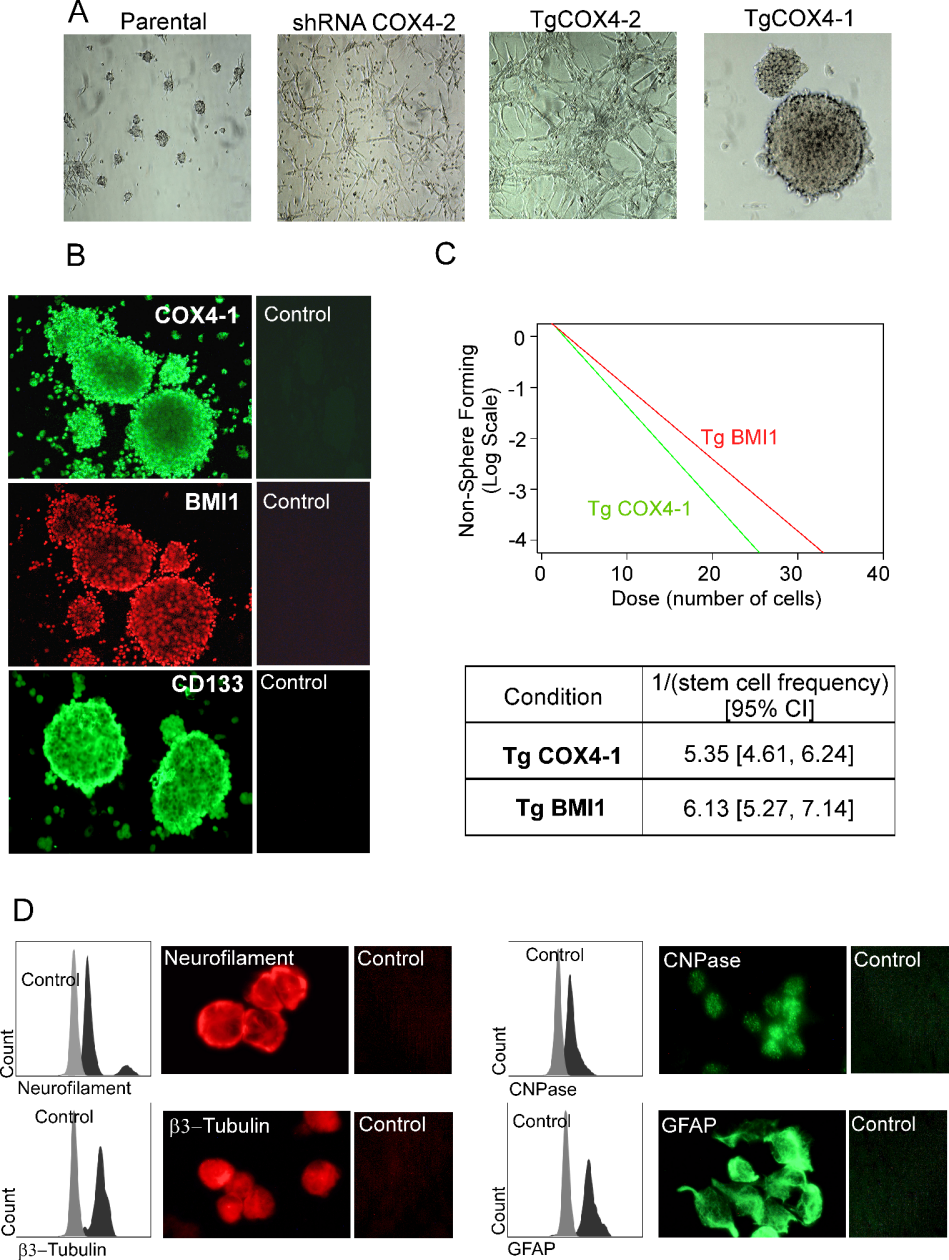
COX4-1 glioma cells form neurosphere-like tumor spheroids expressing neural stem cell markers **(A)** Representative phase contrast photomicrographs (10× magnification) of parental U251, U251-shRNA-COX4-2, U251-TgCOX4-1, and U251-TgCOX4-2 cells after 10 days of culture in serum-free Neurobasal medium supplemented with EGF and FGF. **(B)** Spheroids of U251-TgCOX4-1 cells were immunostained with antibodies against COX4-1, BMI1, or CD133 or with control antibodies. **(C)** *In vitro* limiting dilution assays and quantification of COX4-1 and BMI1 expressing cells. Results represent the average from two independent experiments. **(D)** Spheroid multipotency was assessed by immunofluorescence for neuronal (neurofilament, CNPase, and βIII-tubulin) and glial (GFAP) markers.

Because *BMI1* is a stem cell gene involved in regulation of glioma cell stemness [23, 24], we investigated whether U251-TgCOX4-1 cells are enriched in glioma stem cells (GSCs) when cultured in defined serum-free culture medium supplemented with epidermal growth factor (EGF) and basic fibroblast growth factor (bFGF) (Figure 7A). Only U251-TgCOX4-1 cells formed neurospheres ranging from 0.1 to 1 mm over the course of 72 h. Parental U251 cells formed few and small neurospheres. Interestingly, U251-TgCOX4-2 and U251-shRNA-COX4-2 cells did not form neurospheres and attached to the bottom of the culture dish. To further investigate the effects of COX4-1 on stemness, U251-TgCOX4-1 neurospheres were immunostained for classical stem cell markers CD133, nestin, and BMI1 (Figure 7B). Neurospheres were positive for COX4-1, CD133, and BMI1 but not nestin. Furthermore, when plated in an *in vitro* limiting dilution assay, overexpression of COX4-1 and BMI1 promoted the formation of tumor neurospheres Figure 7C. In agreement with our previous results (Figure 7A), however, COX4-2 expression blocked neurosphere formation. We next induced neurosphere differentiation to determine whether U251-TgCOX4-1 cells are capable of multilineage differentiation. After differentiation with 10% FBS for 7 days, immunocytochemistry was performed using antibodies for neuron-specific β III-tubulin, neurofilament-L, and CNPase and astrocyte-specific GFAP. As illustrated in Figure 7D, U251-TgCOX4-1 cells exhibited immunoreactivity for all neuronal markers tested, indicating multilineage properties. Taken together, these data indicate that COX4-1 promotes the self-renewal capacity of glioma stem-like cells.

## DISCUSSION

CcO is an important mitochondrial multiprotein complex with two main substrates – O_2_ and cytochrome c. The function of CcO as an electron carrier is well documented; however, its role in tumor development and progression is mainly unknown. Previous studies demonstrated that CcO is critically involved in establishing resistance to apoptosis in cervical cancer cells [5, 25] and gliomas [3, 4]. Moreover, we previously showed that acquisition of TMZ-resistance in glioma cells is associated with a significant increase of CcO activity and overexpression of the CcO regulatory subunit COX4-1 [4]. Furthermore, our previous study revealed that CcO is a novel prognostic biomarker in GBM [9]. Our findings here demonstrate that COX4-1 determines proliferative capacity and tumor growth in human glioma cells. Deficiency in COX4-1 reduces CcO activity and mitochondrial function and enhances the accumulation of cellular and mitochondrial ROS, demonstrating that COX4-1 expression is required for maintenance of mitochondrial integrity, as well as for ROS homeostasis in cells.

It has been suggested that COX4-subunit switching provides a mechanism to maintain the efficiency of respiration under conditions of reduced O_2_ availability and may be the initial adaptive response to hypoxia [26]. Additionally, it has been shown that COX4-1, as a key regulatory subunit of mammalian CcO, has an important role in adjusting energy production to match the cellular energy requirements of cancer cells [27, 28]. Although there is evidence suggesting cancer cell proliferation is fueled primarily by a shift to anaerobic glycolysis, a unique metabolic state known as the Warburg effect [29, 30], these observations are challenging to resolve in light of the frequently impaired nutrient availability for cancer cells. Thus, despite the fact that concepts for cancer cell metabolism identified by Warburg have undergone substantial revisions over the last 90 years, the advantage that metabolic transformation confers to cancer cells remains unclear [31, 32]. Indeed, recent studies have revealed that the metabolic characteristics of glioma cells are not as uniform as initially thought, and metabolic heterogeneity and specific differences in glucose uptake and dependency have been demonstrated in glioma cell lines [3, 4, 33–35]. Furthermore, genetically diverse human glioblastomas that exhibited a high rate of glucose uptake were found to use either glycolysis or mitochondrial glucose oxidation as an energy source [36]. Experimental evidence also suggests that differentiation status might correlate with glucose dependency, with glioma stem cells being less reliant on glycolysis than their differentiated counterparts [37]. Given this information, the most direct interpretation is that modulations in the expression of a CcO regulatory subunit such as COX4-1 are related to energy metabolism and redox homeostasis by the cells. It is therefore possible that these changes characterize an aspect of the pleiotropic response of the cells to progression signals or, alternatively, are fundamental in initiating these processes.

It has been previously shown that CcO containing COX4i2 is about twice as active as CcO containing COX4i1 [38]. The discrepancy with our results may be due to cell type-specific responses, differences in normal and cancer cells, or variations in experimental conditions. Specifically, the results reported by Hüttemann et al. [38] reflect the activity of CcO in cow normal tissues (lung, heart, and liver), whereas our study characterized CcO from human brain cancer cells. Additionally, Hüttemann et al. evaluated CcO activity using isolated enzymes [38], whereas we used mitochondrial fractions in which all mitochondrial complexes are present. This is particularly important because the mitochondrial respiratory chain is organized in an array of supercomplexes that operate as one component, which allows more efficient electron transfer between complex III and CcO by cytochrome c, thereby restricting ROS generation [39, 40]. Indeed, mutations in subunits of one ETC complex have been shown to affect the stability of other complexes [38, 39]. Thus, we can speculate that the activity of purified CcO may differ from the activity CcO displays in association with the other complexes of the ETC, a more physiologically relevant condition. Since one major function of supercomplex formation appears to be the limitation of ROS production [39, 40], we further speculate that supercomplex formation may increase ETC coupling, decrease mitochondrial proton leak, and decrease the generation of mitochondrial ROS in CcO carrying the COX4-1 isoform. This may be particularly relevant as ROS are primarily generated at complexes I and III of the ETC, with CcO not normally directly involved in ROS generation. Similar results have been described in oxygen-tolerant HeLa cells, in which a tighter coupling of the ETC due to higher CcO activity depletes upstream electron-rich intermediates responsible for ROS generation [25].

All of these changes together would be expected to contribute to increased tumor cell survival: increased ETC coupling would provide cancer cells with a more efficient energy production; decreased release of cytochrome c minimizing intrinsic activation of apoptosis and decreased ROS would minimize the effects of chemotherapy and perhaps radiotherapy.

Our results showed that COX4-1 expression dramatically increases *BMI1* expression at the mRNA and protein levels. In addition, a significant direct correlation between the expression of *COX4I1* and *BMI1* was observed in human tissue from primary gliomas at the mRNA and protein levels. Furthermore, higher COX4-1 expression correlated significantly with worse patient prognosis, whereas higher COX4-2 expression did not, suggesting that COX4-1 may have a novel function that is different from that of COX4-2. Indeed, our analyses have allowed us to demonstrate that BMI1 is a novel downstream target of COX4-1 and that BMI1 and COX4-1 function cooperatively to regulate the capacity of glioma cancer cells for self-renewal and tumorigenicity.

At present, very little is known about the signaling pathways that regulate the expression of BMI1. Our data suggest that COX4-1 regulates BMI1 expression. Because both COX4i1 and BMI1 are transcriptional targets of c-Myc, our results led us to speculate that BMI1 and COX4i1 may auto-regulate their expression via a positive feedback loop involving c-Myc. This feedback loop regulating BMI1/COX4i1 expression may be relevant in promoting cancer and maintaining stem cell phenotype.

BMI1 is a Polycomb group epigenetic gene silencer that is highly expressed in various types of human cancers [40–42]. It was recently reported that BMI1 expression correlates with poor prognosis and glioma progression in patients [43, 44]. This result is consistent with our findings that BMI1 is a functional target of COX4-1. Furthermore, it was reported that BMI1 has a role in neural stem cell self-renewal [45, 46]. There is substantial evidence that the signaling pathways that regulate cancer stem cell-like properties are similar to those that govern neural stem cell stemness. In addition, it is now recognized that the development of stem cell-like properties in glioma contributes to disease progression [47]. We observed a significant increase in the self-renewing capacity, expression of stem cell markers, and proliferative capacity in glioma cells stably overexpressing COX4-1, suggesting that COX4-1 regulates glioma stem-like cells in part by regulating BMI1 expression.

Multifocal GBM is suggestive of a more proliferative and invasive tumor phenotype, a feature more common to stem cell–derived cancer [48]. Patients with multicentric lesions fare the worst, with average survival of 3 months [49]. It has been suggested that multifocal GBM lesions are the consequence of migratory tumorigenic daughter cells from human brain subventricular tumor stem cells [50–52]. In the present study, we found that U251-TgCOX4-1 cells developed as multicentric lesion tumors, while parental, COX4-2–bearing U251 cells developed tumors as solitary lesions. This result is important because it shows that COX4-1 is essential to drive the overall histopathology of GBM. Prior studies have reported incidences of multiple lesions at the time of GBM diagnosis ranging from 30%–35% [49, 50, 53–55]. Interestingly, we previously detected high CcO activity in about 30% of analyzed GBM samples, and found this higher activity to be an independent prognostic factor for shorter PFS and OS [9]. In the current study, high COX4-1 expression was detected in 8 patients (33%) and was also associated with lower OS. Although we have not fully explored the mechanisms regulating the development of multicentric lesions, our findings suggesting mitochondrial respiration is upregulated in COX4-1-expressing glioma cells rule out the possibility that tumor metabolism is confined to aerobic glycolysis during aggressive growth, in particular in GBMs with multicentric foci.

In conclusion, our data suggest that the utilization of mitochondrial oxidation during aggressive tumor growth may be an adaptive advantage that ensures access to nutrient- and O_2_-rich environments in the brain. We speculate that tumors in this population may represent a novel primary GBM subtype characterized by less intratumoral heterogeneity, increased COX4-1 expression and OXPHOS metabolism, and resistance to stress insults, including radio- and chemotherapies [3, 4, 9].

## METHODS

### Acquisition of tissue specimens

The protocol for this study was approved by the Institutional Review Board for Human Use at the University of Alabama at Birmingham (UAB) (IRB #X050415007). All patients provided written informed consent to the surgical procedures and gave permission for the use of resected tissue specimens. Frozen glioma tissue specimens (24 samples) were obtained from the collection of clinical specimens in the UAB Brain Tumor Tissue Bank from patients who underwent surgical treatment at the UAB Hospital between January 2001 and November 2011.

### Cell culture and electroporation

Glioma cells were cultured as we previously described [3, 4, 34]. All electroporations were performed using a Gene Pulser Xcell Electroporation System (Bio Rad, Hercules, CA) using the following conditions: square wave pulse, 25 msec, and 140V. Four unique 29mer shRNA BMI1-human constructs in the pRFP-C-RS vector (catalog #TF314462) and shRNA COX4-2-human constructs in the untagged pRS vector (catalog #TR305257) were purchased from OriGene Technologies (Rockville, MD). Plasmids with scrambled sequence cassettes were used as negative controls (OriGene Technologies; Catalog # TR30015 and TR30012). COX4-2- and BMI1-stable knockdown cell lines were obtained by selection of puromycin-resistant clones. The stable lines isolated were characterized for the level of COX4-2 and BMI1 by western blot analysis. U251 COX4-2-KO cells were generated using a CompoZr® Knockout ZFN Kit (Sigma-Aldrich, St. Louis, MO) according to manufacturer instructions. U251 COX4-2-KO or -knockdown cells were electroporated with CMV6 plasmids containing FLAG-epitope-tagged COX4-2 or COX4-1 (Catalog # RC209204 and RC209374, OriGene Technologies). To generate stable cell lines overexpressing COX4-1 or COX4-2, cells were selected with G418 for 2 weeks. The stable lines isolated were characterized for the level of mitochondrial COX4-1 and COX4-2 by western blot analysis.

### Mitochondrial preparation and functional studies

Mitochondrial fractions were prepared from cultured cells and human tissue as we previously described [3, 4, 9]. Glucose uptake experiments were carried out as previously described [3, 4] using 2-(N-(7-nitrobenz-2-oxa-1,3-diazol-4-yl)amino)-2-deoxyglucose (2-NBDG; Catalog # N13195, from Life Technologies, Grand Island, NY). Intracellular ROS production was determined by measuring the levels of O2^−^ and H_2_O_2_ produced in the cells by flow cytometry after staining the cells with DCFDA (C-369, Molecular Probes, Grand Island, NY) or MitoSOX™ Red (M36008, Molecular Probes, Grand Island, NY) as we previously described [3]. Mitochondrial complex activities were determined as previously described (3–5). All activities were normalized to citrate synthase activity.

### Cell proliferation and anchorage-independent clonogenic assays

For cell proliferation, glioma cells were seeded into 24-well plates (3 × 10^4^ cells/well). Cell number was counted every 24 h for 4 days. Anchorage-independent clonogenic assays were performed as we previously described [56].

### *In vitro* limiting dilution assay

*In vitro* dilution assays were performed as previously described [58]. Briefly, U251, U251-TgCOX4-1, U251-TgCOX4-2, and U251 cells overexpressing BMI1 were plated at 1, 2, 5, 10, 20, and 40 cells per well in 96-well plates. Ten days after plating, the number of neurospheres in each well and the percentage of positive wells were quantified by manual counting. Extreme limiting dilution assay analyses (ELDAs) were performed on the data as previously described [59].

### Western blot analysis

Western blot analysis was performed as we previously described [3, 4, 34, 57]. The following antibodies were used: anti-citrate synthase (1:1000 dilution, 16131-1-AP, ProteinTech Group, Chicago, IL); anti-DDK (1:2000 dilution, TA50011-5, OriGene Technologies); anti-actin (1:5000 dilution, A1978, Sigma-Aldrich); and anti-BMI1 (1:1000, 6964, Cell Signaling, Beverly, MA). Primary antibodies against COX4-1 (1:1000 dilution, ab14744, Abcam, Cambridge, MA) and COX4-2 (1:1000 dilution, 11463-1-AP, ProteinTech Group) were tested for specificity, and no cross-reactivity between isoforms was detected.

### Animal studies

All surgical and experimental procedures and animal care were performed in accordance and compliance with the policies approved by the University of Alabama at Birmingham Institutional Animal Care and Use Committee (APN 131209529). Confluent human glioma cells were trypsinized to a single-cell suspension, resuspended in PBS and 2 × 10^6^ cells in 0.5ml were subcutaneously injected into the backs of 6-week-old female nude mice. Seven days later, developing tumors were measured in three dimensions. Tumor dimensions were measured twice every week, and tumor volumes were calculated. Mice were killed at 4 weeks after tumor induction, tumors were excised and their *ex vivo* weight and volume measured. Tumors sections were fixed in 4% buffered formaldehyde and processed for histologic examination. Establishment of intracranial tumors was performed as we previously described [57, 58]. Briefly, the scalp of anesthetized athymic nude mice was sanitized with 3 applications of chlorhexidine scrub, a 0.5cm incision made, a 0.45 mm burrhole drilled in the calvarium at 1.0–1.5 mm lateral from the sagittal suture and 2.0 mm anterior to Bregma. A 30G ^½^-inch needle fitted to a 250 μL Hamilton syringe (LT-1725) mounted in a QSI Nanoliter injector attached vertically in a Kopf stereotaxy was inserted 3 mm into the brain. Five μL of cell suspension (1 × 10^8^ cells/ml) was injected at 2.5 μL/min. The needle was withdrawn, the burrhole filled with sterile bonewax and the incision approximated and closed with Tissu-Mend glue. Mice were allowed to recover and were monitored for signs of neurological deterioration at which point they were killed and the brains removed for examination.

### Immunocytochemistry

Paraffin-embedded tumor tissues were serially sectioned (5 μm; CMBD Core Laboratory, UAB), deparaffinized, and rehydrated through a graded ethanol series. To block endogenous peroxidase, the slides were treated with 0.3% hydrogen peroxide in methanol for 20 min. Antigen retrieval was achieved by incubation in citrate-based antigen unmasking solution, pH 6.0 (Vector Labs. Inc., CA) at 95^°^C for 20 min. All subsequent steps were performed using UltraVision Quanto Detection System HRP DAB (Thermo Scientific) according the manufacturer's instructions. Slides were blocked and then incubated overnight at 4°C with the following primary antibodies at a 1:50 dilution: Anti-Ki67 (RM-9106, Thermo Scientific); anti-nestin (4760, Cell Signaling), and anti-β3-tubulin (5568, Cell Signaling). The sections were counterstained with hematoxylin, dehydrated, incubated in xylene, and mounted with Permount (Fisher Scientific). Negative control tissues were treated in the same way, but incubated only with primary antibody or only with secondary antibody. Immunocytochemical staining was performed as we previously described [56].

### Statistical analysis

Data were analyzed using the two-tailed Student t test. Statistical differences were considered significant at *p* < 0.05. Experiments were performed with triplicate samples and were performed twice or more to verify the results.
